# Construction and Evaluation of Recombinant Attenuated *Edwardsiella piscicida* Vaccine (RAEV) Vector System Encoding *Ichthyophthirius multifiliis* (Ich) Antigen IAG52B

**DOI:** 10.3389/fimmu.2021.802760

**Published:** 2022-01-25

**Authors:** Banikalyan Swain, Cole T. Powell, Roy Curtiss

**Affiliations:** Department of Infectious Diseases & Immunology, College of Veterinary Medicine, University of Florida, Gainesville, FL, United States

**Keywords:** fish vaccine, immunity, pathogenicity, *E. piscicida*, *Ichthyophthirius multifiliis* (Ich), innate & adaptive immune response

## Abstract

We have successfully designed and constructed a RAEV vector system with regulated-delayed attenuation *in vivo* attributes that synthesizes *Ichthyophthirius multifiliis* (Ich) protective antigen IAG52B to enable vaccination of fish susceptible to edwardsiellosis and white spot disease. The first feature of this vaccine delivery system is an *Edwardsiella piscicida* strain carrying genomic deletions of *asdA*. AsdA is an enzyme necessary for the synthesis of diaminopimelic acid (DAP), which is an essential component of the peptidoglycan layer of the cell wall of Gram-negative bacteria. *asdA* mutant strains have obligate growth requirements for DAP in the medium or a plasmid vector with the wild-type *asdA* gene enabling synthesis of DAP. This balanced-lethal plasmid vector-host system in *E. piscicida* enables as a second feature the synthesis of recombinant antigens to induce protective immunity against fish pathogens. Recombinant protective antigen IAG52B from the fish pathogen *I. multifiliis* was synthesized by RAEV strains harboring the AsdA^+^ plasmid pG8R8029. The third feature of this vaccine strain is a regulated-delayed attenuation *in vivo* phenotype that is based on the replacement of an arabinose-regulated *araC* P_araBAD_ cassette for the promoters of the *fur* and *crp* genes of *E. piscicida* such that the expression of these genes is dependent on arabinose provided during growth. Thus, following colonization, the Fur and Crp proteins stop being synthesized due to the lack of arabinose and attenuation is progressively achieved *in vivo* to prevent generation of diseases symptoms. Our vaccine strain χ16022 with the genotype Δ*asdA10* ΔP_fur170_::TT *araC* P_araBAD_ *fur* ΔP_crp68_::TT *araC* P_araBAD_ *crp* contains the AsdA^+^ plasmid, pG8R8029, which encodes the IAG52B antigen. Vaccine strain χ16022(pG8R8029) is attenuated and induces systemic and mucosal IgM titer against *E. piscicida* and Ich in zebrafish. In addition, transcript levels of *tnf-α*, *il-1β*, *il-6* and *il-8* were significantly increased in different tissues of vaccinated zebrafish compared to unimmunized fish. Zebrafish vaccinated with χ16022(pG8R8029) showed 60% survival upon intracoelomic (i.c.) challenge with a lethal dose of virulent *E. piscicida* strain J118. Our RAEV system could be used as a generalized vaccine-vector system to protect teleost fish against multiple bacterial, viral and parasitic infectious diseases.

## 1 Introduction

*Edwardsiella piscicida* is a Gram-negative, facultative anaerobic bacterium of the family *Enterobacteriaceae*. It is the causative agent of edwardsiellos in many economically important species of fish including catfish (*Ictalurus furtatus*), tilapia (*Tilapia nilotica*), European eel (*Anguilla anguilla*) and Indian major carp (*Catla catla)* (1–3). Vaccination would be an effective method to prevent and control *Edwardsiella* outbreaks. Live-recombinant attenuated bacterial vaccines must be fully attenuated to prevent disease symptoms and highly immunogenic. Conventional in-frame gene deletion can over attenuate the strain, making it more susceptible to host immune defenses and limit its ability to colonize internal tissue (4). To overcome these problems, our lab has previously demonstrated the ability to effectively attenuate *E. piscicida* through the use of regulated-delayed attenuation systems (5, 6). The promoters of the *fur* and *crp* genes were replaced by a tightly-regulated *araC* P_araBAD_ cassette such that expression was arabinose dependent during growth *in vitro.* This feature enables the strain to phenotypically mimic the virulent of wild-type *E. piscicida* strain at the time of vaccination and enhance the tissue colonization. Inside host tissues, absence of arabinose prevents the synthesis of the Fur and Crp proteins, allowing attenuation to gradually manifest and prevent disease symptoms (4). The safety and control of these strains can be further enhanced by replacing the promoters of several virulence factors with externally-regulated promoters. Additionally, these recombinant attenuated *Edwardsiella* vaccines (RAEVs) are capable of stimulating both innate and adaptive immune responses in fish (5, 6). Zebrafish vaccinated with an RAEV showed up-regulation in the transcription of genes for several pro-inflammatory cytokines, including TNF-α and IL-1β (5, 7). Additionally, immunized fish had significantly higher levels of serum IgM and increased levels of protection against challenge with the wild-type strain (5). These attributes suggest that these RAEV strains would be optimal delivery systems for heterologous recombinant proteins.

The ability of an organism to act as a recombinant protein producer is contingent on its ability to effectively retain the expression vector. To ensure that the vector is not lost, a selective pressure is created so that only organisms harboring the vector with the selective marker are able to grow. In laboratory settings, antibiotics typically serve as the selective agent. However, vectors with antibiotic selection markers may not be retained *in vivo* when the selective pressure is removed. Thus, vectors used in live-attenuated vaccines should have a selective marker that is applicable in host tissue. Diaminopimelic acid (DAP) is a necessary precursor for peptidoglycan synthesis in Gram-negative bacteria (8). Aspartate ß-semialdehyde dehydrogenase is an enzyme required for the production of DAP and encoded for by the *asdA* gene (9, 10). Gram-negative bacteria lacking *asdA* are unable to synthesize DAP and undergo lysis if they cannot obtain it from their surroundings (11–14). DAP is not synthesized nor metabolized in mammalian tissue and the nutritional requirements of fish suggests they also lack biological pathways that utilize DAP as a substrate (15, 16). This makes *asdA* an appropriate selection marker for live vaccines and is the basis for the balanced-lethal system, in which *asdA* deficient bacteria are dependent on a vector containing the cloned wild-type *asdA* gene for survival (17). This system is utilized in many Recombinant Attenuated Salmonella Vaccines (RASVs) to deliver protective antigens against multiple pathogens through antigen secretion or surface display (18, 19). Additionally, *E. ictaluri* and *E. tarda* Δ*asdA* mutants are capable of expressing heterologous antigens harbored on AsdA^+^ vectors (14, 20). These findings suggest that an RAEV possessing an *asdA* deletion could also function in a same manner.

*Ichthyopthirius multifiliis* is a ciliated protozoan and the etiological agent of white spot disease in fish. Infection by *I. multifiliis* has been shown to elicit various primary and secondary immune responses in catfish that promote parasite clearance (21–24). These adaptive immune responses in fish suggests that vaccination would be a safe and effective method to prevent disease. Surface immobilization antigens (IAGs) expressed by *I. multifiliis* are immuno-dominant and have become candidate proteins for vaccine development (23). Channel catfish and rainbow trout immunized with DNA vaccines encoding IAG52B produced specific antibodies, but showed no significant increase in the level of protection in challenge studies, suggesting that naked DNA alone is insufficient to induce a strong enough immune response to confer protection (25, 26).

The purpose of this study was to construct an antibiotic-sensitive and highly immunogenic Recombinant Attenuated *Edwardsiella* Vaccine (RAEV) vector system with regulated-delayed attenuation *in vivo* attributes. The *asdA* gene was deleted and an optimal balanced-lethal system was developed by using diverse recombinant AsdA^+^ plasmids containing different promoter sequences and origins of replication to generate as a platform for the heterologous antigen delivery system. The *I. multifiliis* gene encoding the surface immobilization antigen 52B (IAG52B) was codon optimized to enable high-level expression in *Edwardsiella*. In addition, a codon specifying incorporation of glutamine in *Edwardsiella* was specified to replace the nonsense codon used to specify glutamine in *I. multifiliis*. This modified sequence was then inserted into an AsdA^+^ expression vector and fused with the β-lactamase type II signal sequence for better secretion of the cloned gene products into the periplasm. The strain was then evaluated for its ability to elicit innate and adaptive immune responses towards both *E. piscicida* and *I. multifiliis* in immunized zebrafish.

## 2 Materials and Methods

### 2.1 Bacterial Strains, Plasmids, Media, and Reagents

All bacterial strains and plasmids used in this study are listed in **Table 1**. Bacterial strains were grown on Luria-Bertani (LB) agar or in LB broth. Where necessary, media was supplemented with 15% agar, 10% sucrose, colistin sulfate (Col) (12.5 µg/ml), chloramphenicol (Cm) (25 µg/ml), diaminopimelic acid (DAP) 50 µg/ml. Growth of bacteria was determined by spectrophotometrically and/or by plating following serial dilution. Oligonucleotides were from IDT (Coralville, IA). New England BioLabs restriction endonucleases and T4 ligase were used for cloning. For all PCR reactions, GoTaq DNA polymerase (Promega, catalog# M3008) was used. For plasmid DNA isolation and purification of gel fragments and PCR products, Qiagen products (Hilden, Germany) were used.

**Table 1:** Bacterial strains and plasmids.

Strain or Plasmid	Genotype/Relevant Characteristics	Source or Reference(s)
***E. coli* Strains**		
χ7213	*thi-1 thr-1 leuB6 glnV44 fhuA21 lacY1 recA1* RP4-2-Tc::Mu *λpir ΔasdA4 Δzhf-2*::Tn*10*	(27)
χ6212	F*^-^* Λ*^-^* φ80 Δ(*lacZYA-argF*) *endA1 recA1 hsdR17 deoR thi-1 glnV44* *gyrA96 relA1* Δ*asdA4*	(17)
***E. piscicida* Strains**		
J118	Wild-type *E. piscicida* EIB202, highly virulent, fish isolated, Col^r^	(28)
χ16000	Δ*asdA10*	This study
χ16010	ΔP_crp68_::TT *araC* P_araBAD_ *crp*	(6)
χ16012	ΔP_fur170_::TT *araC* P_araBAD_ *fur*	(5)
χ16015	Δ*asdA10* ΔP_fur170_::TT *araC* P_araBAD_ *fur*	This study
χ16022	Δ*asdA10* ΔP_fur170_::TT *araC* P_araBAD_ *fur* ΔP_crp68_::TT *araC* P_araBAD_ *crp*	This study
**Plasmids**		
pRE112	Suicide vector; *sacB mob*RP4 R6K *ori*; Cm^r^	(29)
pG8R8000	Δ*asdA10*, pRE112	This study
pG8R8009	ΔP_crp68_::TT *araC* P_araBAD_ *crp*, pRE112	(6)
pG8R8024	ΔP_fur170_::TT *araC* P_araBAD_ *fur*, pRE112	(5)
pG8R8011	3095 bp, *E. piscicida* P_asdA_ *asdA*, pUC ori	This study
pG8R8012	3001 bp, *E. piscicida* SD *asdA*, pUC ori	This study
pG8R8013	2983 bp, *E. piscicida* ATG-*asdA*, pUC ori	This study
pG8R8014	3001 bp, *E. piscicida* SD-GTG-*asdA*, pUC *ori*	This study
pG8R8015	3095 bp, *E. piscicida* P_asdA_ *asdA*, pBR *ori*	This study
pG8R8016	3001 bp, *E. piscicida* SD *asdA*, pBR *ori*	This study
pG8R8017	2983 bp, *E. piscicida* ATG-*asdA*, pBR *ori*	This study
pG8R8018	3001 bp, *E. piscicida* SD-GTG-*asdA*, pBR *ori*	This study
pYA3493	*Salmonella* AsdA^+^ vector, 3113 bp, pBR *ori* β-lactamase signal sequence-based periplasmic N-terminal sequence secretion plasmid	(30)
pG8R8029	1269 bp of codon-optimized IAG52B ORF was cloned into the *EcoRI* and *BamHI* site of pYA3493	This study
pYA3341	*Salmonella* AsdA^+^ vector, 2595 bp, pUC *ori*	(30)
pYA3342	Salmonella AsdA^+^ vector, SD *asdA* gene. pBR *ori*	(30)
pYA3332	*Salmonella* AsdA^+^, p15A *ori*	(31)
pYA3337	*Salmonella* AsdA^+^, pSC101 *ori*	(32)
pEZ142	*E. ictaluri* AsdA^+^ vector, Cm, pACYC184 p15 *ori*	(14)

### 2.2 Experimental Animals

Adult zebrafish, *Danio rerio* (2 ± 0.5 cm and 0.4 ± 0.05 g) deemed clinically healthy were acclimated to laboratory conditions for two weeks after purchase from Aquatic Research Organisms, Hampton, NH, U.S.A. Conditioned reverse osmosis (RO) water was used in the zebrafish cultivation system. Water temperature was maintained at 26°C with a conductivity between 300-400 µS and pH between 7.0 and 7.4 by adding instant sea salt and sodium bicarbonate. Water was changed every day. A 14/10 h light/dark cycle was utilized, with lights turned on at 7:00 am and off at 9:00 pm. Fish were fed commercial zebrafish feed, GP 500-800 Micron Weaning Diet (Brine shrimp direct) two times per day. Zebrafish were anaesthetized by immersion in 100 ng/ml of tricaine methanesulphonate (MS-222) (Tricaine-S, Syndel, USA). Before manipulations, fish were euthanized for at least 10 min with Tris-buffered MS-222 at 20 mg/lit.

### 2.3 Sequence Analysis

Publicly available AsdA protein sequences were retrieved from NCBI GenBank database. *E. piscicida* (CP001135), *E. hoshinae *(WP_024522689), *E. ictaluri* (WP_015872886), *E. anguillarum* (WP_034163973.1), *E. coli* (AP_004358), *S.* Gallinarum (WP_000799940), *Salmonella* Paratyphi A (ATF61156.1), *E. tarda* (WP_109728620), *A. hydrophila* (ABK39477.1), *A. salmonicida* (WP_005310917), *Vibrio* (WP_001263690), *S.* Typhimurium (AKH09169). *S. flexneri* (YP_690789), and *Yersiniaceae* (WP_120132887.1) sequences were used to construct the unrooted phylogenetic tree of AsdA by the neighbor-joining method of MEGA6 program (33). The three-dimensional (3D) structures of the *E. piscicida*, *E. ictaluri* and *S*. Typhimurium AsdA protein was predicted by using Phyre2 web portal (http://www.sbg.bio.ic.ac.uk/phyre2/html/page.cgi?id=index).

### 2.4 Construction of RAEV Strains

#### 2.4.1 Construction of *asdA* Mutants

To develop antibiotic-sensitive strains of live-attenuated recombinant bacterial vaccines, a balanced-lethal host-vector system was constructed by deletion of the aspartate β-semialdehyde dehydrogenase (*asdA*) gene. The *ΔasdA10* defined deletion mutation encompasses a 1100 base pair deletion including the ATG start codon but not does not include the last four bases, “CTAG”, specifying the stop codon for the gene. Primers asdA-1F-XbaI and asdA-2R (**Table 2**) were designed to amplify the upstream of the *asdA* gene flanking region (432 bp). The downstream *asdA* gene flanking region (583 bp) was amplified by primers asdA-3F and asdA-4R-KpnI (**Table 2**). A *Xba*I site was included in asdA-1F-XbaI, and a *Kpn*I site was included in asdA-4R-KpnI. The flanking regions were amplified from *E. piscicida* J118 genomic DNA. The two PCR fragments were joined by overlapping PCR with primers asdA-1F-XbaI and asdA-4R-KpnI, and the products were cloned into the *Xba*I/*Kpn*I site of the suicide vector pRE112 (29). The resulting plasmid was designated pG8R8000. To construct the *E. piscicida ΔasdA10* mutant, the suicide plasmid was transferred from *Escherichia coli* χ7213 to *E. piscicida* wild-type strain J118 through conjugation. LB agar plates containing Col, Cm, and DAP were used to isolate strains with single-crossover plasmid insertions. A sacB-based sucrose sensitivity counter-selection system was used to select for bacteria that had lost the suicide vector after a second homologous recombination (i.e., allelic exchange) (29). The colonies were screened for growth in the presence of DAP, as well as for Cm^S^, and Col^r^. The *ΔasdA*-deletion mutant was confirmed by PCR and DNA sequencing.

**Table 2:** Primers used in this study.

Primer	Sequence (5`-3`)
asdA-1F-XbaI	CATTCTAGATCCGGATATTTCATATAGCTTTCAAT
asdA-2R	CGCGGACTAGATGCACTCCTGCCTTGGATGGTGACGAGTTG
asdA-3F	AGGAGTGCATCTAGTCCGCGCCCTGGTACGGCGCAGGC
asdA-4R-KpnI	CATGGTACCATTTCTTATTTAATGCCCTGAATACC
PasdA-F	CATTCTAGAAAATTCACTTGCGCATCGCGGC
SDasdA*-*F	CATTCTAGATCACCATCCAAGGC**AGGA**GTGCATATG
asdA-F	CATTCTAGAGTGCAT**ATG**AAAAACGTTGGTT
SDasdA-GTG-F	CATTCTAGATCCAAGGCAGGAGTGCAT**GTG**
ASD-RV	CATGGTACCGACTAGAGCAGCAGCCTCAGC
p42F-KpnI	CATGGTACCAGACCTTCCATTCTGAAATGA
p42R-XbaI	CATTCTAGACTGTCAGACCAAGTT
TNF-α-F	AAGGAGAGTTGCCTTTACCG
TNF-α-R	ATTGCCCTGGGTCTTATGG
IL-1β F	TGGACTTCGCAGCACAAAATG
IL-1β R	CACTTCACGCTCTTGGATGA
IL-6 F	TCAACTTCTCCAGCGTGATG
IL-6 R	TCTTTCCCTCTTTTCCTCCTG
IL-8 F	GTCGCTGCATTGAAACAGAA
IL-8 R	CTTAACCCATGGAGCAGAGG
β-actin-F	CCGTGACATCAAGGAGAAGCT
β-actin-R	TCGTGGATACCGCAAGATTCC

Restriction enzyme sites were underlined. Start codon and SD sequences were in bold letter.

#### 2.4.2 Construction of RAEV Strains With Regulated Delayed Attenuation Phenotype

To construct the *E. piscicida ΔasdA10* strain (χ16000) with a regulated-delayed attenuation phenotype, *Δ*P_fur_ and *Δ*P_crp_ deletion insertion mutations were added sequentially to χ16000 by using suicide plasmids pG8R8024 and pG8R8009 as described previously (5, 6). The resultant strain had a genotype Δ*asdA10* ΔP_fur170_::TT *araC* P_araBAD_ *fur* ΔP_crp68_::TT *araC* P_araBAD_ *crp* and numbered as χ16022.

### 2.5 Construction of AsdA^+^ Plasmids for Complementation of *E. piscicida *Δ*asdA* Mutant Strains

A series of different AsdA^+^ plasmid vectors were constructed with pUC ori and pBR *ori* containing the *E. piscicida asdA* gene with modifications of the *asdA* promoter, SD sequence and start codon. The *E. piscicida asdA* gene was amplified with its wild-type promoter and Shine-Dalgarno (SD) sequence, or with only the SD *asdA* sequence and also with modification of the start codon from ATG to GTG or the *asdA* gene without its SD sequence by using primers listed in **Table 2**. Forward and reverse primers were tagged with restriction enzyme sites for *Xba*I and *Kpn*I. Fragments of the pYA3341 (pUC *ori*) (30) and pYA3342 (pBR *ori*) plasmids (30) minus the *S.* Typhimurium *asdA* gene were amplified by PCR with the primer pair P42F-KpnI and p42R-XbaI (**Table 2**). After gel purification, fragments were ligated with T4 DNA ligase and transformed into the *E. piscicida* Δ*asdA* strain χ16000 and plated on LB agar plates. The recombinant plasmids were confirmed by restriction digestion with *Xba*I and *Kpn*I and sequencing. The resulting plasmids were named pG8R8011, pG8R8012, pG8R8013, pG8R8014, pG8R8015, pG8R8016, pG8R8017 and pG8R8018 (**Table 1**).

### 2.6 Growth Curve Analysis

The growth of the *E. piscicida* Δ*asdA* mutant strain harboring different AsdA^+^ vectors was analyzed and compared to the mutant strain without a plasmid in LB both in presence and absence of DAP. A series of plasmids were constructed with differing copy numbers, presence or absence of the promoter for the *asdA* gene and with either ATG or GTG start codons as described in **Table 1**. Standing overnight 30°C cultures (OD_600_ ~ 0.6) of *E. piscicida* strains were diluted 1:100 into prewarmed LB or LB plus DAP broth and incubated at 30°C with shaking at 180 RPM. The OD_600_ was measured every 60 min. The growth curves were calculated using the automated growth curve device Bioscreen C (Growth Curves USA, Piscataway, NJ).

### 2.7 Determination of Lethal Dose 50 (LD_50_)

To determine the LD_50_ of *E. piscicida* strains i.e. J118, χ16000, χ16010, χ16012 and χ16012(pYA3493) ten-fold serial dilutions of *E. piscicida* fresh cultures were made in sterile BSG, and the concentration of bacteria was determined by the spread-plate method. Fish were i.c. injected in a dose of 10 µl of BSG containing different concentrations of CFU/fish (**Table 3**) with 10 fish in each group (two replicate tanks, 5 fish in each tank). Mortality was documented daily over a 15-day period, and the Reed and Muench method was used to calculate the LD_50_ values (34).

**Table 3:** LD_50_ study of *E. piscicida* wild-type and vaccine strains.

J118 (Wild-Type *E. piscicida*)									
Dose	Mortality	Deaths	Survivals	Accumulated			Mortality Ratio	Mortality %	LD_50_
				Death	Survival	Total			
**2 × 10^3^**	1/10	1	9	1	13	14	1/14	7	1.1×10^4^
**2 × 10^4^**	7/10	7	3	8	4	12	8/12	67
**2 × 10^5^**	9/10	9	1	17	1	18	17/18	94
**2 × 10^6^**	10/10	10	0	27	0	27	27/27	100
**χ16000** (Δ*asdA10*)									
**3 × 10^4^**	0/10	0	10	0	29	29	0	0	7.5×10^6^
**3 × 10^5^**	0/10	0	10	0	19	19	0	0
**3 × 10^6^**	4/10	4	6	4	9	13	4/13	31
**3 × 10^7^**	7/10	7	3	11	3	14	11/14	78
**χ16010** (ΔP_crp68_::TT *araC* P_araBAD_ *crp*)									
**2 × 10^3^**	0/10	0	10	0	25	25	0	0	1.8×10^5^
**2 × 10^4^**	3/10	3	7	3	15	18	3/18	17
**2 × 10^5^**	4/10	4	6	7	8	15	7/15	47
**2 × 10^6^**	8/10	8	2	15	2	17	15/17	88
**2 × 10^7^**	10/10	10	0	25	0	25	25/25	100
**χ16012 (**ΔP_fur170_::TT *araC* P_araBAD_ *fur* **)**									
**2 × 10^3^**	0/10	0	10	0	16	16	0	0	2×10^4^
**2 × 10^4^**	6/10	6	4	6	6	12	6/12	50
**2 × 10^5^**	8/10	8	2	14	2	16	14/16	87
**2 × 10^6^**	10/10	10	0	24	0	24	24/24	100
**χ16022** (Δ*asdA10* ΔP_fur170_::TT *araC* P_araBAD_ *fur* ΔP_crp68_::TT *araC* P_araBAD_ *crp*)									
**5 × 10^4^**	0/10	0	10	0	30	29	0	0	1.4×10^7^
**5 × 10^5^**	1/10	1	9	1	20	19	1/19	5
**5 × 10^6^**	3/10	3	7	4	11	13	4/13	31
**5 × 10^7^**	6/10	6	4	10	4	14	10/14	71
**χ16022** (**pYA3493) **(Δ*asdA10* ΔP_fur170_::TT *araC* P_araBAD_ *fur* ΔP_crp68_::TT *araC* P_araBAD_ *crp*)									
**1.5 × 10^4^**	0/10	0	10	0	17	17	0	0	2×10^5^
**1.5 × 10^5^**	6/10	6	4	6	7	13	6/13	46
**1.5 × 10^6^**	7/10	7	3	13	3	16	13/16	81
**1.5 × 10^7^**	10/10	10	0	23	0	23	23/23	100

### 2.8 Colonization of RAEV Strain in Zebrafish

Zebrafish were inoculated with ~5 x 10^3^ cells of χ16022, carrying the Ich IAG52B antigen gene, in a dose of 10 µl of BSG by i.c. injection. The spleen and kidneys were collected at days 3 and 5 after inoculation. Tissues were homogenized in 200 µL of BSG. A 10-fold serial dilution of each sample was used and plated on LB agar plates containing 0.2% arabinose and 10 µg/mL of colistin sulfate. The plates were incubated at 30˚C for 48 h and then the colonies were counted to determine the bacterial load in each organ. The results obtained from five fish (n = 5) in each time point were plotted with the bars indicating standard error.

### 2.9 Immune Protection Against Wild-Type *E. piscicida* Challenge

The immune protection mediated by χ16022 against wild-type *E. piscicida* challenge was evaluated following i.c. vaccination, with a dose of 1 x 10^4^ cells/fish using 15 fish/group. Fish were given a booster dose of 1 x 10^4^ cells/fish after 2 weeks. Control fish were injected with 10 µL of BSG. Two weeks after receiving the booster dose, fish were challenged by i.c injection of virulent, wild-type *E. piscicida* J118 at a dose of 1 x 10^5^ cells/fish. Mortalities were recorded for 14 days and represented as percent survival. The experiment was repeated once using 15 fish/group. Since the results from the two independent experiments were similar, the data were pooled together and represented as 30 fish total.

### 2.10 Subcellular Fractionation

The periplasmic fraction was prepared by following the method described by Kang et al., 2002 (30). *E. piscicida* and *E. coli* cells harboring the pG8R8029 plasmid were grown in LB broth at 30˚C or 37˚C up to OD_600_ of 0.8 and centrifuged at 6000 g for 10 min. The supernatant fluid was saved for analysis of secreted proteins. Equal volumes of periplasmic, cytoplasmic, outer membrane, supernatant fractions and total lysate samples were separated by SDS-PAGE for western blot analysis.

### 2.11 SDS-PAGE and Western Blotting

Plasmid encoding IAG52B (pG8R8029) and control plasmid (pYA3493) were electroporated into *E. piscicida* or *E. ictaluri* or *E. coli* cells. Strains were grown in LB broth and 0.2% arabinose was added when necessary. Bacterial cells were grown at 30°C or 37°C with aeration (180 rpm) to an optical density at 600 nm (OD_600_) of 0.8. For the western blot analysis, 1 ml of bacterial culture was centrifuged, suspended in 100 µl of phosphate buffered saline (PBS; pH 7.4) and mixed with 100 µl of 2X SDS loading buffer. Protein samples were boiled for 10 minutes, and then 10 µl samples were loaded onto a 12% SDS-polyacrylamide gel electrophoresis (SDS-PAGE) gels and electrophoresed. Samples were transferred onto nitrocellulose membranes. Membranes were blocked overnight at 4°C using fat-free milk powder dissolved in phosphate buffered saline (PBS) (5%, wt/vol) supplemented with 0.05% Tween 20 (PBS-T). The membranes were incubated with a primary rabbit polyclonal anti-Ich antibody (35). Membranes were washed with PBS-T three times, and then incubated with an alkaline phosphatase-conjugated anti-rabbit immunoglobulin G (IgG) (Sigma) diluted 1:10,000 in blocking buffer at room temperature for 1 h. A mixture of nitroblue tetrazolium and 5-bromo-4-chloro-3-indolylphosphate (NBT-BCIP) (Amaresco), chromo-genic substrates for alkaline phosphatase was used to develop color. The reaction was stopped after 10 min by washing with several large volumes of deionized water.

### 2.12 RNA Isolation and First-Strand cDNA Synthesis

Total RNA was extracted from zebrafish tissue samples (i.e., gill, kidney, intestine, and spleen) using TRIzol^®^(Ambion) following the standard protocol (Invitrogen). The total RNA concentration was measured by a UV-spectrophotometer (NanoDrop2000c, Thermo), and the relative purity was analyzed by the ratio of the absorbance value at 260 nm (A260) divided by the absorbance value at 280 nm, with a value of ~2.0 being considered highly pure. 3 µg of total RNA was then treated with 1 U of Thermo Scientific™ DNase I, RNase-free (FEREN0521) to remove any residual DNA from the sample. Reverse transcription was carried out using oligo-dT primer and Thermo Scientific™ RevertAid™ Premium First Strand cDNA Synthesis Kit (Catalog #FERK1622). cDNA synthesis was confirmed by PCR amplification of the ß-actin gene, keeping DNase-treated RNA as a negative control. Until further analysis, the synthesized cDNA was stored at -80˚C.

### 2.13 Quantitative Real-Time PCR (qRT-PCR) Analysis

To evaluate the ability of χ16022 specifying the Ich IAG52B antigen to stimulate an immune response, the expression of the genes encoding IL-1β, IL-6, IL-8, and TNF-α in zebrafish tissues was analyzed by qRT-PCR. A 10 µL reaction mixture was prepared consisting of 5 µL of 2 x PowerUp™ SYBR™ Green Master Mix (Thermofisher Catalog # A25742), 3.5 µL of PCR grade H_2_O, 0.25 µL of FW and RV primers (2.5 mM each), and 1.0 µL of cDNA. The reaction was carried out in a Quantstudio 3 thermocycler (Applied Biosystems) in three separate wells with conditions of initial denaturation at 95°C for 10 min followed by 45 cycles with denaturation at 94°C for 10 s, annealing at 58°C for 10 s and extension at 72°C for 10 s. The β-actin gene was used for the internal normalization and reaction mixtures without the cDNA template served as the negative controls. PCR efficiencies were determined by analyzing cDNA serial dilutions. Since the efficiencies were almost 100%, the 2^−ΔΔCT^ method (36) could be used to calculate the relative gene expression compared to the B-actin gene reference. The relative expression ratios were obtained by normalizing expression of the target gene, as determined by mean crossing point (cp) deviation by that of B-actin. The single band amplification and correct size was confirmed by ethidium bromide-stained 1% agarose gels using 8 µL of the qRT-PCR product. The qRT-PCR data was expressed as a mean of three individual experiments with standard error. To determine the significant difference between control and treated groups, Student’s t-test was conducted using Microsoft Excel 2010 with a significance level of *P* < 0.05.

### 2.14 Determination of IgM by ELISA

ELISA was used to assay antibodies in gill, skin and serum to *E. piscicida* LPS and Ich membrane protein. Samples were prepared as described previously (5). Polystyrene 96-well flat-bottom microtiter plates (Dynatech Laboratories Inc., Chantilly, Va.) were coated with *E. piscicida* LPS or Ich membrane protein (100 ng/well), in sodium carbonate-bicarbonate coating buffer (pH 9.6) 100 μl volumes in each well. The coated plates were incubated for an overnight at 4°C. Free binding sites were blocked with 5% bovine serum albumin (BSA) for 1 h at room temperature. After washing, 100-μl volume of diluted zebrafish anti-serum/mucus sample was added to individual wells in duplicate and incubated for 2 h at 37°C. The plates were treated with mouse anti-zebrafish IgM monoclonal antibody (Aquatic Diagnostics Ltd) for 1 h at room temperature. Plates were then incubated with biotinylated goat anti-mouse IgG (Southern Biotechnology Associates, Birmingham, AL) for 1 h at room temperature. After incubation of wells with a streptavidin-alkaline phosphatase conjugate (Southern Biotechnology) for 1 h at 37°C, *p*-nitrophenyl phosphate (PNPP, Thermo Fisher Scientific) was added for color development. The optical density (OD) units were read at 405 nm using an automated ELISA plate reader (model EL311SX; Biotek, Winooski, VT).

### 2.15 Statistical Analysis

Statistical analysis was performed by GraphPad Prism 6 (Graph Pad Software, Inc., San Diego, CA, USA). Survival data was analyzed with the log-rank (Mantel-Cox) test. Differences between the groups were analyzed by two-way ANOVA, where asterisks (*) indicate a significant difference (*P < 0.05, **P < 0.01, ***P < 0.001).

## 3 Results

### 3.1 Sequence Analysis, Phylogenetic Tree and 3D Model of AsdA

**Figure 1:**
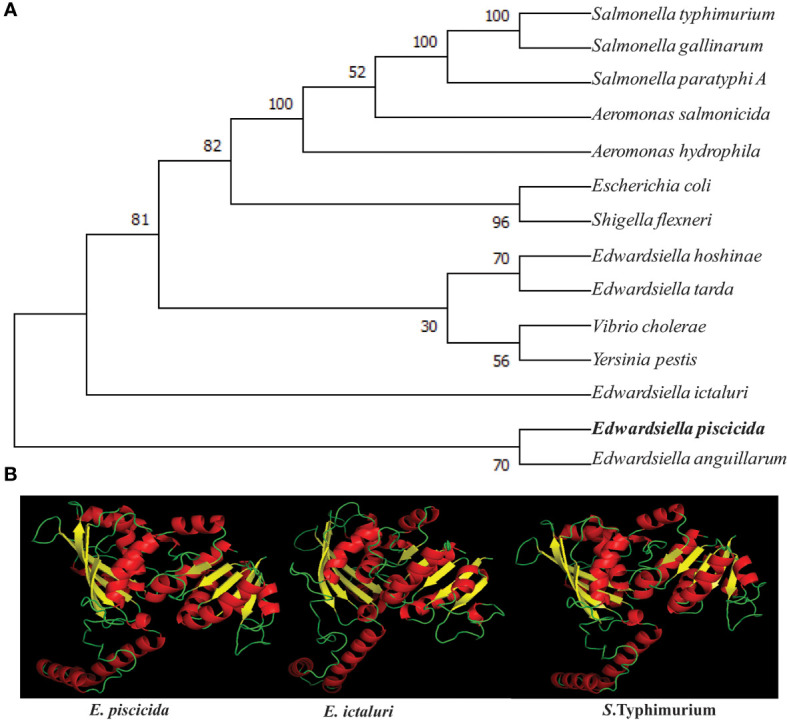
Phylogenetic and structural similarity of *E piscicida* AsdA with other bacteria. **(A)** Amino acid sequences of AsdA of various bacteria were retrieved from the GenBank database and the unrooted phylogenetic tree of AsdA was constructed by the neighbor-joining method within the MEGAX program. **(B)** 3D structure of *E*. *piscicida*, *E*. *ictaluri* and *S*. Typhimurium AsdA proteins were predicted by using (PS)2: Protein Structure Prediction Server Version 3.0 web portal.

The *E. piscicida* EIB202 (J118) *asdA* open reading frame consisted of 1104 base pairs (bp) that encoded a putative 368 amino acid (aa) residue protein with an estimated molecular mass of 40 kilodaltons (kDa). To explore the evolutionary development history of bacterial *asdA* genes, a phylogenetic tree was constructed based on the amino acid sequences (**Figure 1A**). The amino acid sequence of *E. piscicida* AsdA shares a high percentage of sequence identity with *E. anguillarum* (98.64%), *E. ictaluri* (97.28%), *E. tarda* (94.02%) and *E. hoshinae* (92.92%). These sequence identities were reflected in the phylogenetic tree. *E. piscicida* and *E. anguillarum* form a cluster together with a high bootstrap value. *E. ictaluri* forms a separate but close cluster to *E. piscicida*. *E. hoshinae* and *E. tarda* fall in same cluster and were separated from other *Edwardsiella* species. *Salmonella* Typhimurium, *Salmonella* Gallinarum and *Salmonella* Paratyphi A shared 82.83% of sequence identity with *E. piscicida*.

The 3D structure of the *E. piscicida, E. ictaluri* and *S.* Typhimurium AsdA were predicted by using the Phyre2 web portal. Our results revealed that they share high sequence homology and with similar 3D structures. Each protein showed 16 α-helices and 9 β-sheets that were connected to each other by loops (**Figure 1B**).

### 3.2 Construction and Characterization of *E. piscicida asdA* Mutant

**Figure 2:**
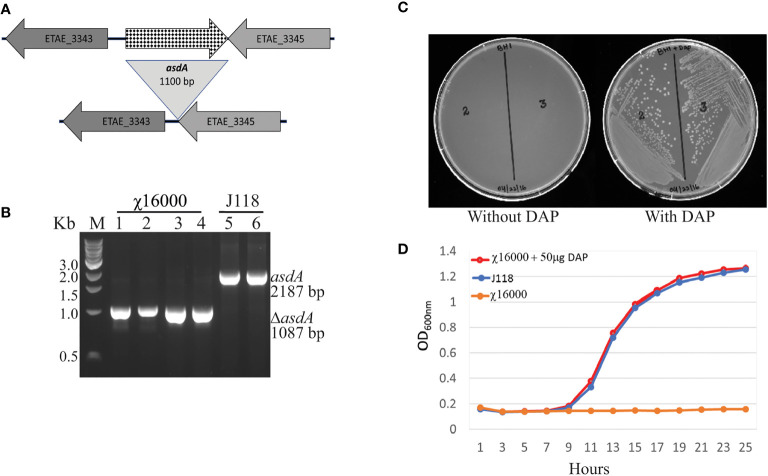
*(E) piscicida asdA* (χ16000) genotype and phenotype verification. **(A)** Deletion map of Δ*asdA*. **(B)** Genotype verification of *E. piscicida ΔasdA* (χ16000) by PCR. **(C)** Phenotypic verification of Δ*asdA* (χ16000) in the presence or absence of DAP in LB agar plates. **(D)** Growth curves for J118 and χ16000 in presence or absence of DAP.

To develop antibiotic-sensitive strains of live-attenuated recombinant bacterial vaccines, we used a balanced-lethal host-vector system to delete the aspartate β-semialdehyde dehydrogenase (*asdA*) gene. The *ΔasdA10* defined deletion mutation encompasses a 1100 base pair deletion including the ATG start codon but does not include the last four bases, “CTAG”, of the gene. The upper panel of **Figure 2A** illustrates the chromosomal structures of the wild-type and mutant strains. Construction of the *E. piscicida* Δ*asdA* strain was done through suicide plasmid mediated homologous recombination, using suicide vector pG8R8000, a pR112 (Cm) based suicide vector (**Table 1**), by allelic replacement in the parent strain J118. The genotype was confirmed *via* PCR (**Figure 1B**) and sequencing. Agarose gel analysis and sequencing of PCR product confirmed that Δ*asdA* was 1100 bp smaller than the wild-type strain. The resultant *E. piscicida* Δ*asdA* mutant was named χ16000 (**Table 1**). The phenotype was verified by growth of Δ*asdA* mutant (χ16000;) in presence or absence of DAP (**Figures 2C, D**). The Δ*asdA* mutant could not survive in LB agar plates without being supplemented with DAP, but in the presence of DAP, the Δ*asdA* mutant strain grew like the wild-type strain. These results confirm that the *E. piscicida asdA* gene is functional and might work in a similar manner to that observed in *E. ictaluri* (14) and *Salmonella* (17).

### 3.3 Construction of *E. piscicida* Vaccine Delivery Strains With Regulated-Delayed Attenuation Phenotype

Deletion of either *fur* or *crp* attenuated *E. piscicida* (5, 6) and *E. ictaluri* (37, 38). Strains were constructed with deletion insertion mutations that conferred a phenotype of regulated-delayed attenuation *in vivo* (5). The principle of regulated-delayed attenuation *in vivo* is based on the replacement of the *fur* and *crp* gene promoters with a tightly regulated *araC* P_araBAD_ cassette such that the expression of these genes is dependent on arabinose supplied during growth. Thus, following internal tissue colonization, Fur and Crp protein synthesis is terminated due to the absence of arabinose and attenuation is steadily manifested *in vivo* to prevent disease symptoms. The promoters, including all sequences that interact with activator or repressor proteins, for the *fur* and *crp* genes were deleted, and the improved *araC* P_araBAD_ cassette was substituted in *E. piscicida* Δ*asdA* strain (χ16000) to yield a strain with genotype Δ*asdA10*; ΔP_fur170_::TT *araC* P_araBAD_ *fur*; ΔP_crp68_::TT *araC* P_araBAD_ *crp* (P stands for promoter and TT for transcription terminator), and the strain was designated χ16022.

### 3.4 Construction AsdA^+^ Vectors to Develop a Balanced-Lethal System in *E*. *piscicida*

Gram-negative bacteria with *asdA* mutants have an obligate requirement for diaminopimelic acid (DAP), which is a crucial component of the peptidoglycan layer (4). In surroundings deprived of DAP, i.e., animal tissues, they will undergo lysis. Deletion of the *asdA* gene has previously been used to develop antibiotic-sensitive strains of live-attenuated recombinant bacterial vaccines (14, 19). Introduction of an AsdA^+^ plasmid into a *ΔasdA* mutant makes the bacterial strain plasmid dependent (17). This dependence on the AsdA^+^ plasmid vector creates a balanced-lethal complementation between the bacterial strain and the recombinant plasmid.

**Figure 3:**
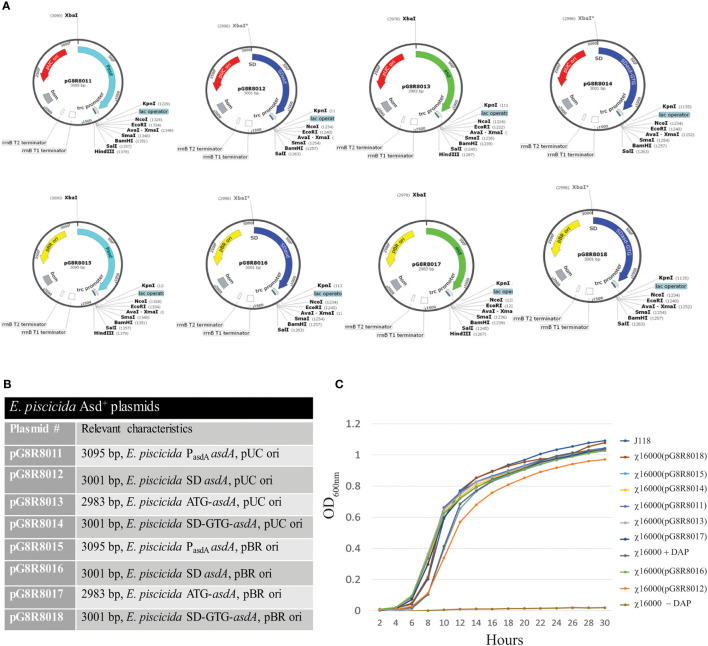
Complementation of Δ*asdA* mutant strains with *E*. *piscicida* AsdA^+^ Plasmids. **(A)** *E*. *piscicida* AsdA^+^ Plasmid maps (pG8R8011-pG8R8018) showing origin of replication, orientation of *asdA* gene, presence or absence of the promoter for the *asdA* gene and with either ATG or GTG start codons, P_trc_ promoter and multiple cloning sites. **(B)** Characteristic of *E*. *piscicida* AsdA^+^ plasmids or various strategies to construct AsdA^+^ plasmids *via* modification of the plasmid copy number (pUC *ori* and pBR *ori*), promoter, SD sequence and start codon. **(C)** Growth of Δ*asdA* (χ16000) mutant strains complemented with different *E*. *piscicida* AsdA^+^ plasmids (pG8R8011 - pG8R8018).

Eight different AsdA^+^ plasmid vectors were constructed with pUC *ori* or pBR *ori* containing the *E. piscicida asdA* gene with modifications of the *asdA* promoter, SD sequence and start codon (**Figures 3A, B**). Plasmids pG8R8011 to pG8R8014 contain the pUC *ori* whereas plasmids pG8R8015 to pG8R8018 have the pBR *ori*. Plasmid pG8R8011 and pG8R8015 contain the wild-type *asdA* gene with the wild-type promoter, which includes the 118 bp upstream sequence from the start codon (ATG). Plasmid pG8R8012 and pG8R8016 contain *E. piscicida asdA* ORF with Shine-Delgrano (SD) sequence “AGGA”. pG8R8013 and pG8R8017 contain only the wild-type *asdA* ORF sequence. Plasmid pG8R8014 and pG8R8018 encode *asdA* sequence with the start codon “GTG” and (SD) sequence “AGGA”. Each of these plasmids were individually incorporated into the Δ*adsA* strain χ16000, and growth curves determined (**Figure 3C**). All of these *E. piscicida* AsdA^+^ plasmids were able to complement the *asdA* gene of χ16000. The strain complemented with pG8R8018, which has the pBR *ori* and encodes the *asdA* sequence with the start codon “GTG” and (SD) sequence “AGGA” grew similar to the wild-type. χ16000 complemented with pG8R8014 grew slightly slower compared to other complemented strains. pG8R8014 and pG8R8018 have similar features other than the origin of replication.

### 3.5 Complementation of *E. piscicida* Δ *asdA* Strains With *Salmonella* and *E. ictaluri* AsdA^+^ Plasmids

**Figure 4:**
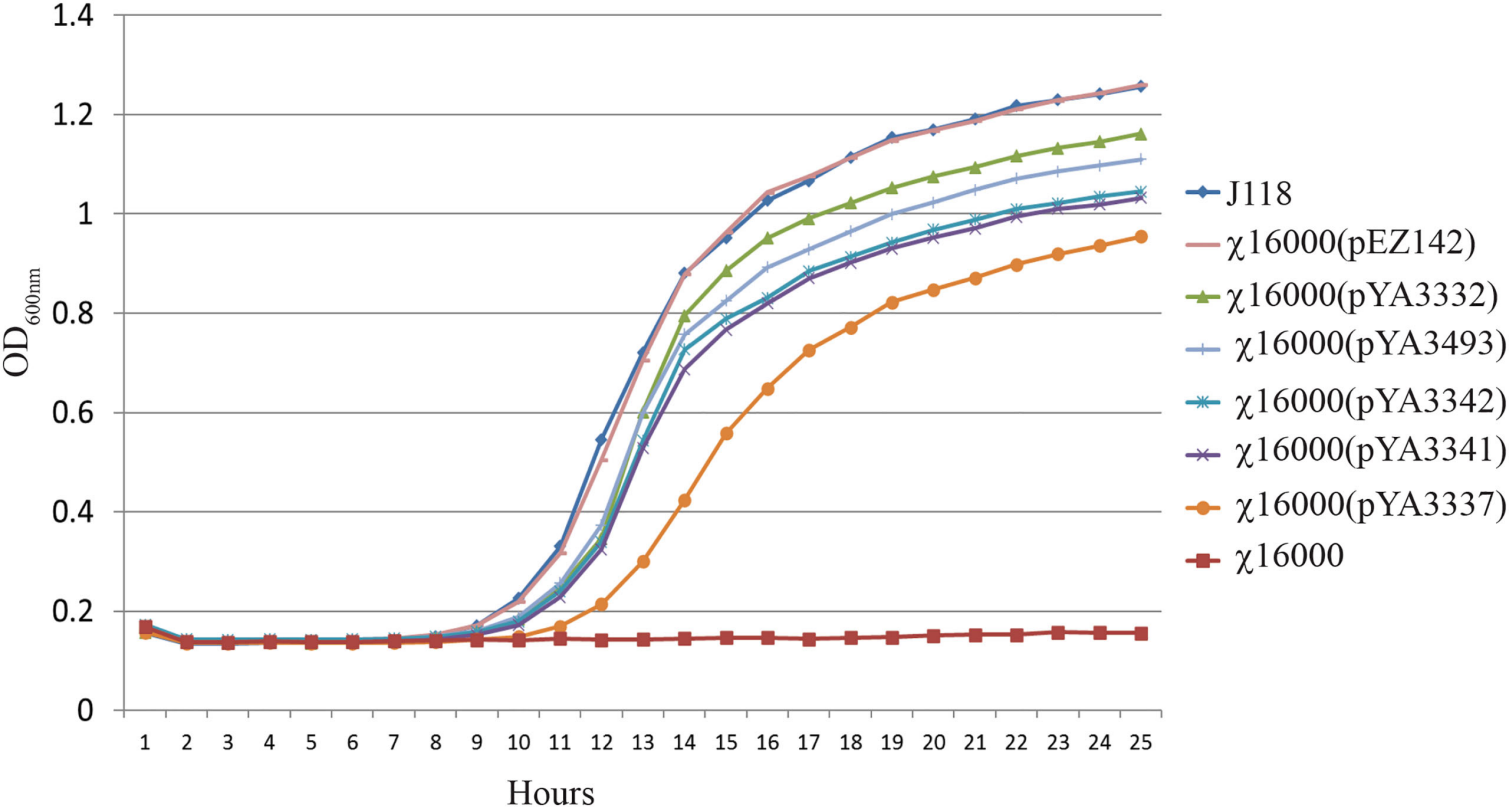
Growth of *E. piscicida* Δ*asdA* (χ16000) strains complemented with *asdA* gene from *E. ictaluri* and *Salmonella.* Growth of *E. piscicida* Δ*asdA* (χ16000) strains complemented with *Salmonell*a AsdA^+^ plasmids with different copy numbers (high, medium and low) and modified *asdA* gene promoter.

The *E. piscicida* AsdA enzyme shares 97% and 81% sequence identity with *E. ictaluri* and *Salmonella*, respectivelly. Therefore, compared the ability of the *asdA* gene from *E. ictaluri* and *Salmonella* to complement the *E. piscicida* Δ*asdA* mutant (**Figure 4**). χ16000 complemented with pEZ142 showed a similar growth curve to wild-type J118. A high (pUC *ori*), medium (pBR & p15A *ori*) and low-copy (pSC101 *ori*) number *Salmonella* AsdA^+^ plasmids were used (**Table 1**) to complement *E. piscicida* Δ*asdA* mutants. All of these plasmids efficiently complemented the Δ*asdA* chromosomal mutation; however, the growth of these strains was slower than those complemented by plasmids with the *E. piscicida* AsdA+ encoding sequences. A slower growth of χ16000(pYA3337) was observed compared to other strains. The slower growth might be due to the low-copy number plasmid pYA3337, which may not produce a sufficient amount of AsdA enzyme needed for the growth of the bacteria.

**Figure 5:**
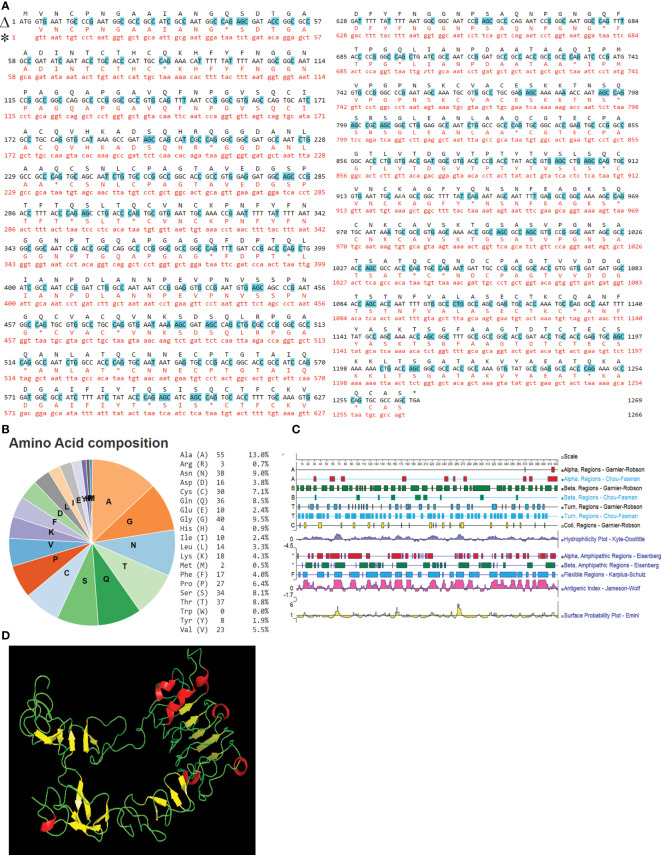
IAG52B sequence analysis. **(A)** Alignment of nucleotide and amino acid sequences of the original and codon-optimized IAG52B gene. Line marked with Δ is the codon optimized sequence and * is the original IAG52B sequence. Changed nucleotide sequences are highlighted with blue. **(B)** Pie chart and table illustrates the amino acid composition of IAG52B. **(C)** The secondary structures of IAG52B protein were predicted by Protean software by using Gramier-Robson and Chou-Fasman methods. Lines 1, 3, 5, and 7 are the Gramier -Robson methods; the red represents the alpha helix, the green represents the beta fold, the blue represents the turn, the yellow represents the random coil. Lines 2, 4, 6 are for the Chou -Fasman method; the red represents the alpha helix, the green represents the beta fold, the blue for the turn, without random coil prediction. **(D)** The 3D structure of IAG52B protein was predicted using the Phyre2 web portal.

IAG52B ORF consisted of 1383 base pairs and encoded a protein of 460 aa. Different domains of IAG52B were predicted by the InterPro web portal (https://www.ebi.ac.uk/interpro/). IAG52B contains a C-terminal signal IP of 20 amino acids, a middle non-cytoplasmic domain of 422 amino acids and a N-terminal transmembrane region of 18 amino acids. Transgene expression of i-antigen genes imposes a challenge in heterologous systems such as bacteria, because Ich, like other hymenostome ciliates, uses a nonstandard genetic code (39–41) in which the stop codons UAA and UGA encode glutamine. This problem was addressed by synthesizing and inserting i-antigen gene constructs with altered codons that were optimized for expression in *E. coli* and *E. piscicida* (**Figure 5A**). The composition of the IAG52B amino acid is depicted in **Figure 5B**. The secondary structure of IAG52B (**Figure 5C**) was predicted by Protean software, and the 3D structure (**Figure 5D**) was predicted by Phyre2 web portal. Our results indicate that the IAG52B protein contains 20 β-sheets and 7 α-helices and is connected by loops.

**Figure 6:**
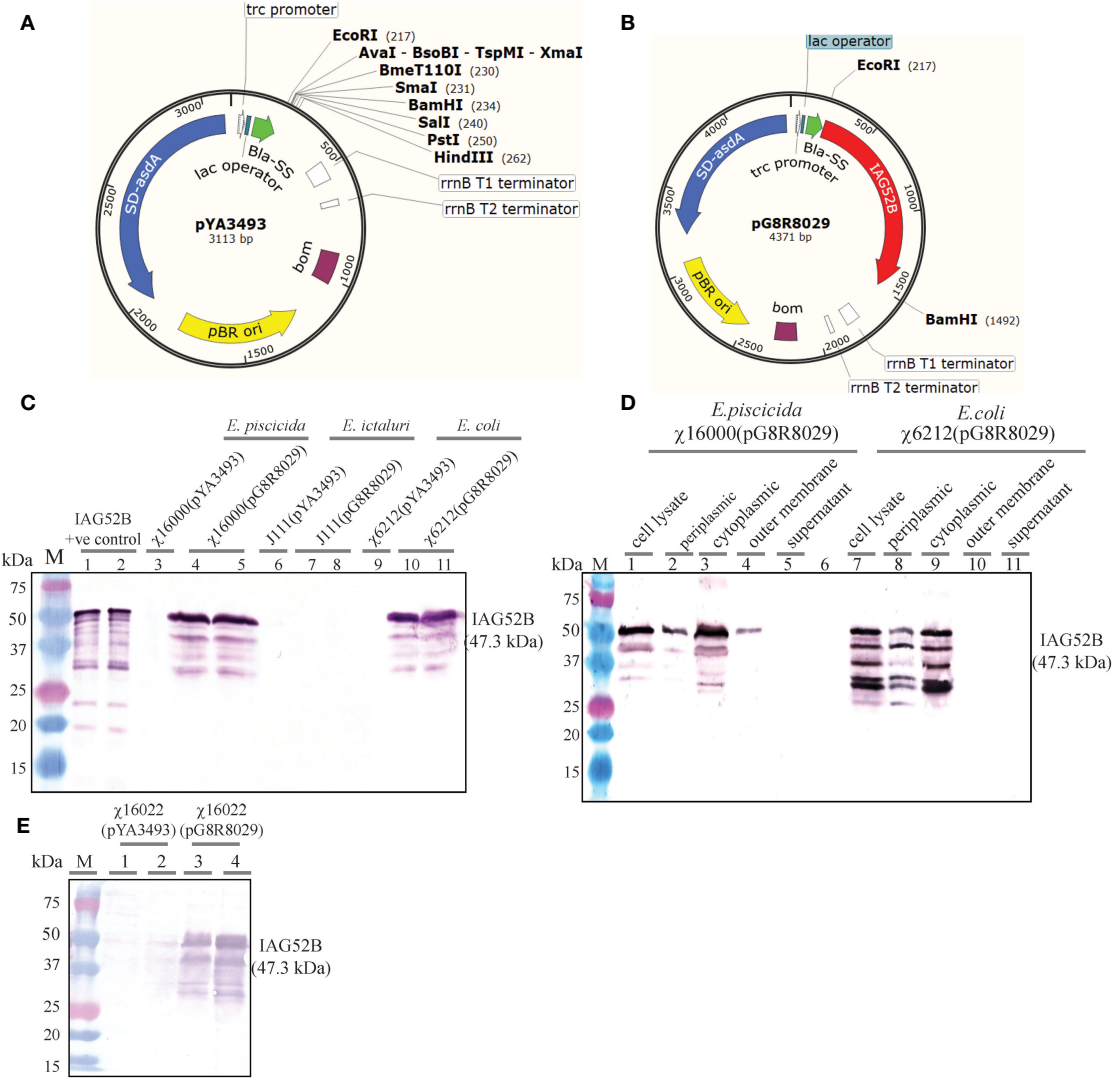
**(A)** Vector map of pYA3493 (pBR *ori*), which is a periplasmic secretion AsdA^+^ vector. A DNA fragment encoding the β-lactamase signal sequence (*bla* SS) and 12 amino acid residues of the N-terminus of mature-lactamase was positioned under the control of the P_trc_ promoter. Map of pYA3493 shows the unique restriction enzyme sites in the multiple cloning site. **(B)** Vector map of recombinant plasmid pG8R8029. Codon-optimized gene of IAG52B non-cytoplasmic domain of 1269 bp was PCR amplified and cloned into the pYA3493 at *Eco*RI-*Bam*HI sites. The IAG52B gene was fused into the same reading frame of *bla* SS and under the control of the P_trc_ promoter. **(C)** Synthesis of IAG52B antigen in *E*. *piscicida*, *E. ictaluri* and *E. coli asdA* mutant strains. pYA3493 (vector control) or pG8R8029 (encoding IAG52B) were electroporated into *E. piscicida* (χ16000) or *E*. *ictaluri* (J111) or *E*. *coli* (χ6212). IAG52B antigen synthesis was analyzed in these cells by western blotting using an anti-IAG52B antibody. **(D)** Subcellular location of synthesized IAG52B in *E*. *piscicida* and *E*. *coli*. Western blot showing IAG52B synthesis in whole cell lysate, periplasmic, cytoplasmic, outer membrane and supernatant fraction of χ16000 and χ6212 harboring pG8R8029. **(E)** Analysis of IAG52B antigen synthesis in *E*. *piscicida* vaccine strain χ16022. pYA3493 (vector control) or pG8R8029 (encoding IAG52B) was electroporated into χ16022, IAG52B antigen synthesis was analyzed by western blotting by using anti-IAG52B antibody.

The codon-optimized sequence of IAG52B non-cytoplasmic domain of 1269 bp was PCR amplified and cloned into the pYA3493 (**Figure 6A**) at the *Eco*RI-*Bam*HI site. The IAG52B gene was fused into the same reading frame of *bla* SS under the P_trc_ promoter. The resultant plasmid was named as pG8R8029 (**Figure 6B**). Both pYA3493 and pG8R8029 were introduced into the Δ*asdA* strains: *E. piscicida* (χ16000), *E. ictaluri* (J111) and *E. coli* (χ6212). The synthesis of IAG52B was analyzed by western blotting. As shown in **Figure 6C**, synthesis of IAG52B was detected in *E. piscicida* and *E. coli*, but no signal was noticed in *E. ictaluri* and cells harboring the control plasmid pYA3493.

*E. piscicida* strain χ16000 and *E. coli* strain χ6212 harboring pG8R8029 were analyzed for subcellular localization of IAG52B. As expected, IAG52B was detectable in cytoplasm, periplasm and the outer membrane in both χ16000(pG8R8029) and χ6212(pG8R8029) (**Figure 6D**). A high amount of protein was detected in the cytoplasmic fraction but only half of the IAG52B was secreted into the periplasmic fraction in both *E. piscicida* and *E. coli*. Interestingly, some of i-antigen associated with the outer membrane in *E. piscicida* was detected*;* this suggests that some antigen may be incorporated into outer membrane vesicle (OMVs) that are very immunogenic and excellent means for antigen delivery. Next, the synthesis of IAG52B in the vaccine delivery strain χ16022 was analyzed. Large amounts of IAG52B synthesis were detected in χ16022(pG8R8029) and no signal was observed in the vector control χ16022(pYA3493) (**Figure 6E**). This result confirms that IAG52B is synthesized in the *E. piscicida* vaccine strain χ16022(pG8R8029). A similar subcellular localization pattern was observed in χ16000(pG8R8029).

To examine the stability of plasmids pYA3493 and pG8R8029 in *E. piscicida* χ16022, strains χ16022(pYA3493) and χ16022(pG8R8029) were cultured with daily passage of 1:1,000 dilutions for five consecutive days in LB broth containing DAP and arabinose. Hundred colonies were screened daily for DAP sensitivity in LB agar plates in the presence or absence of DAP. All the colonies were DAP independent, indicating that pYA3493 and pG8R8029 are very stable in the χ16022 vaccine strain. IAG52B synthesis was analyzed by western blotting from cells obtained from the last day of culture of the stability test, and there was a detectable signal, which suggests both pG8R8029 in the vaccine strain and antigen expression are stable.

### 3.6 Verification of Regulated-Delayed Attenuation of *E. piscicida* Mutant Strains in Zebrafish

The virulence of the *E. piscicida* vaccine and wild-type strains was determined by LD_50_ values in zebrafish injected intracoelomically with different concentrations of bacteria. Our result showed that the vaccine strain χ16022 harboring the control plasmid pYA3493 had significantly higher LD_50_ levels compare to the wild-type strain (**Table 3**). The LD_50_ of the wild-type strain J118 was 1.1 × 10^4^ CFU, while the LD_50_ of the χ16022(pYA3493) increased up to 2×10^5^ CFU, which is more than a 10-fold increase (**Table 3**). As expected, the LD_50_ of the χ16022(pYA3429) was similar to the LD_50_ of χ16022(pYA3493) (data not shown). This result indicates that the *E. piscicida crp* gene product contributes to the virulence and pathogenicity of *E. piscicida*. The LD_50_ of χ16012 was 2 x 10^4^ CFU, which was similar to the wild-type strain J118. This result confirms that ΔP_fur170_::TT *araC* P_araBAD_ *fur* deletion insertion mutation may not affect the virulence of *E. piscicida* by i.c. injection. As expected, the LD_50_ of the χ16022(pYA3429) was similar to the LD_50_ of χ16022(pYA3493) (data not shown).

### 3.7 Dissemination and Colonization of χ16022(pG8R8029) in Zebrafish Tissues

**Figure 7:**
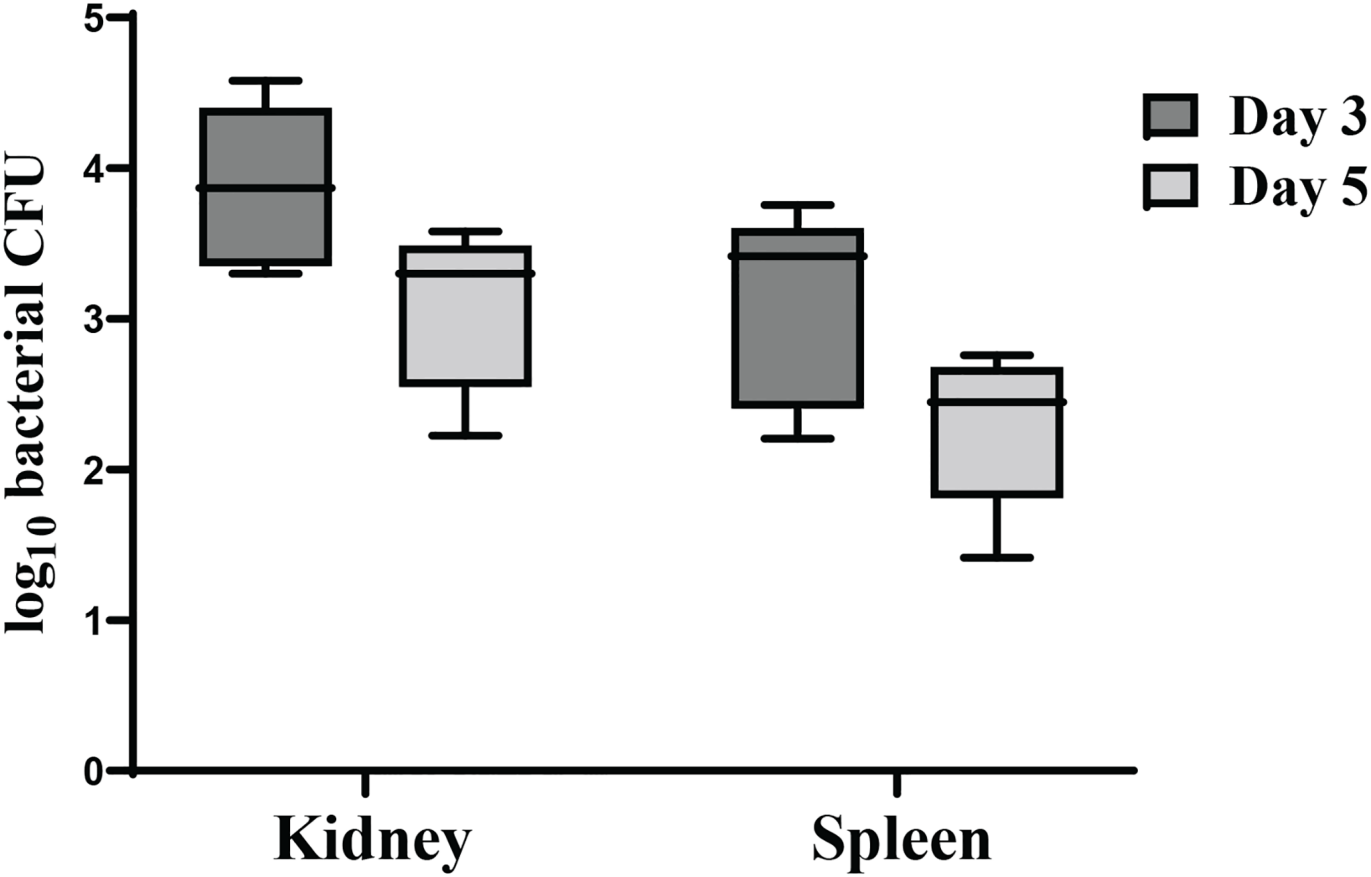
Dissemination and colonization of χ16022(pG8R8029) in zebrafish tissues. **(A)** Zebrafish were vaccinated with χ16022(pG8R8029) by i.c. injection. Kidney and spleen were collected from immunized fish at 3- and 5-days post-vaccination (five fish in each group). The tissues were homogenized in 200 μL of BSG and plated on LB agar plates supplemented with 0.2% agarose and 10 μg/mL of colistin. The plates were incubated at 30°C for 48 h and the colonies were counted. The data was a combination of three independent assays.

To examine the ability of χ16022(pG8R8029) to colonize and disseminate into fish tissues, zebrafish were immunized with the vaccine strain χ16022(pG8R8029) by i.c. injection. The recovery of the vaccine strain from the kidney and spleen was determined at days 3 or 5 post-vaccination. A significant number of χ16022(pG8R8029) was recovered in both the kidney and spleen at days 3 or 5 of post-vaccination. However, the number of bacteria was higher at day 3 compared to day 5 (**Figure 7**). This result indicates that the vaccine strain successfully colonized and disseminated into different tissues of fish.

### 3.8 Assessment of Cytokine Gene Expression in Different Organs of Immunized Zebrafish

**Figure 8:**
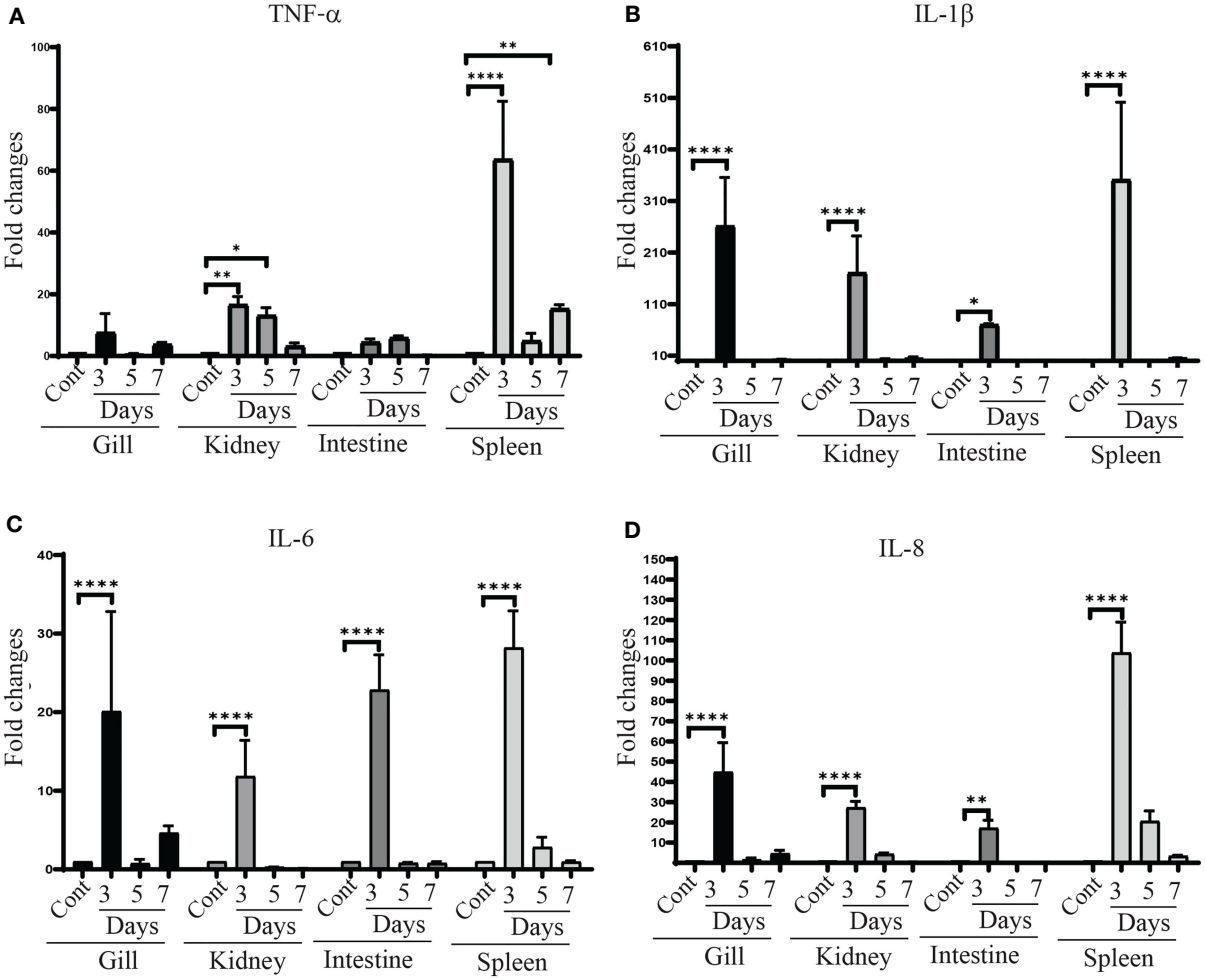
*tnf-α, il-1β, il–6* and *il-8* gene expression in χ16022(pG8R8029) vaccinated and control zebrafish. Zebrafish were vaccinated with χ16022(pG8R8029) by i.c. injection. At three, five and seven days of post-immunization, total RNA was extracted from the gills, kidney, intestine and spleen and cDNA was prepared. The qRT-PCR assay was conducted to analyze the expression of *tnf-α, il-1β*, *il-6* and *il-8* genes using *β-actin* as an internal control. The results are expressed as mean ± standard error (bars) from three separate experiments. Differences between uninfected (control) and infected groups were analyzed by two-way ANOVA, where asterisks (*) indicate significant difference (*P < 0.05, **P < 0.01, ****P < 0.0001) with respect to the control group.

To investigate the efficiency of the vaccine strain in modulating immune responses of zebrafish, the expression of genes associated with the immune response including tumor necrosis factor-α (*tnf-α*), interleukin 1β (*il-1β*), interleukin-6 (*il-6*) and interleukin 8 (*il-8*) in the gills, kidney, intestine and spleen were assessed by qRT-PCR. All of these cytokine genes were significantly upregulated in the tissues of vaccinated fish compared to unvaccinated fish. Furthermore, their expression was higher at day 3 compared to day 5 and 7 in all tested tissues of the vaccinated fish. A significant increase in *tnf-α* gene expression was detected in kidney and spleen (**Figure 8A**). *il-1β* gene expression levels were significantly increased in all organs after 3 days post-vaccination. No changes were found in the expression levels of *il-1β* at day 5 and 7 in vaccinated fish compared to the control group (**Figure 8B**). The expression of *il-6* and *il-8* patterns were similar to *il-1β*, showing significant upregulation in all tissues at day 3, whereas, the changes at day 5 and day 7 were insignificant compared to control tissues (**Figures 8C, D**).

### 3.9 Antibody Responses to *E. piscicida* LPS and Ich Membrane Protein

**Figure 9:**
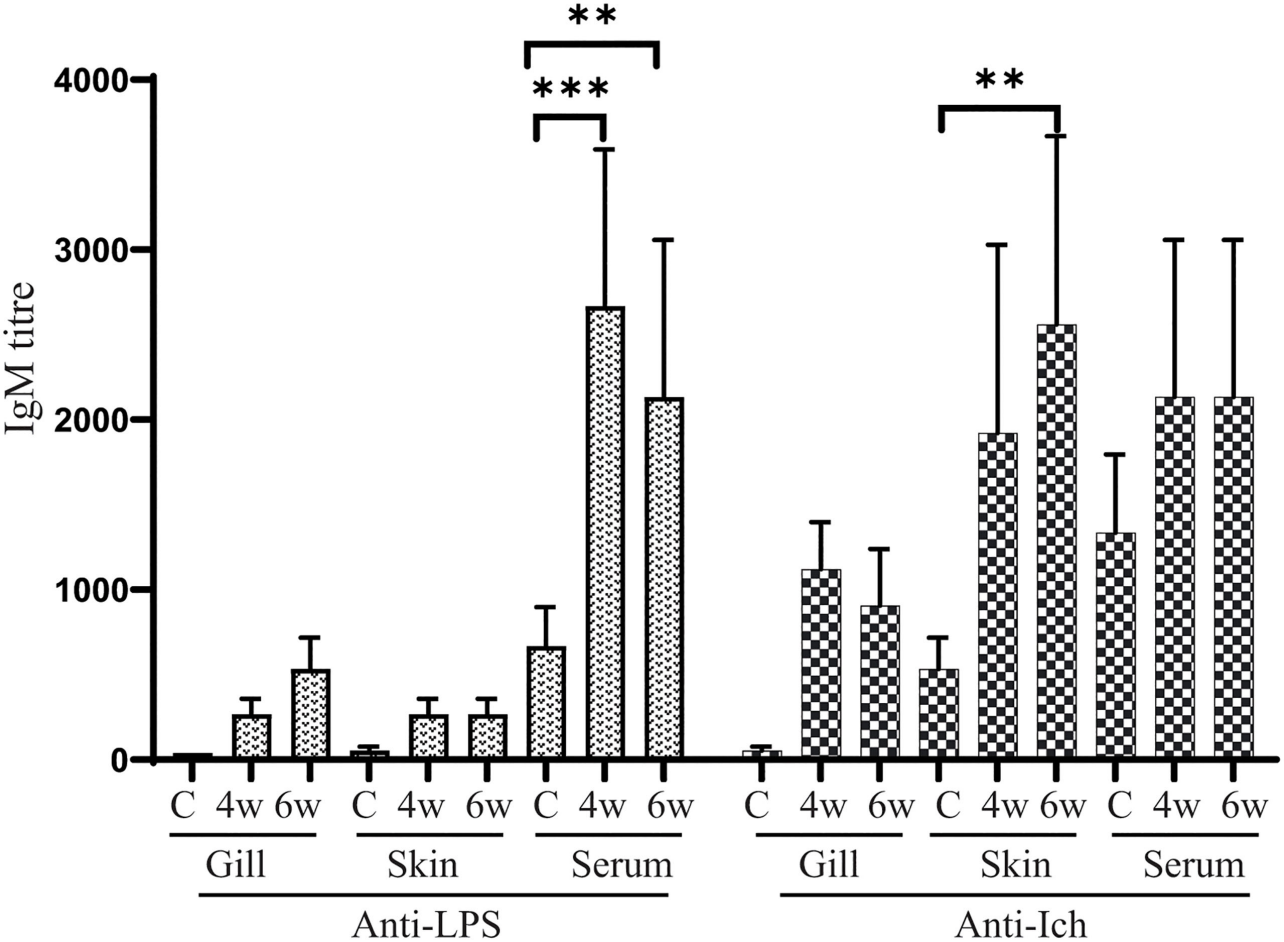
Anti-LPS and anti-Ich antibody responses in zebrafish. Serum and mucosal immunoglobulin M (IgM) responses to *E. piscicida* lipopolysaccharide (LPS) and Ich IAG52B membrane protein were measured by ELISA at 4 weeks (4w) post-vaccination with χ16022(pG8R8029) and 2 weeks after J118 challenge (6w). The results are expressed as mean ± standard error (bars) from three separate experiments. Differences between treated and control groups were analyzed by two-way ANOVA, where asterisks (*) indicate significant difference (**P < 0.01, ***P < 0.001).

Serum and mucosal immunoglobulin M (IgM) responses to *E. piscicida* lipopolysaccharide (LPS) and Ich membrane protein were measured by ELISA at 4 weeks (before challenge) and 6 weeks (2 weeks after challenge) post-vaccination of χ16022(pG8R8029). The LPS and Ich specific IgM titers were induced in both serum and mucosal tissues of vaccinated fish and compared to unvaccinated control fish. There was a significant upregulation of anti-LPS IgM titers in the serum at both 4- and 6-weeks post-vaccination compared to control group. The anti-Ich IgM titer was significantly increased only at 6 weeks of post-vaccination (**Figure 9**).

### 3.10 Immune Protection of Vaccine Strain χ16022(pG8R8029) Against Virulent *E. piscicida* in Zebrafish

**Figure 10:**
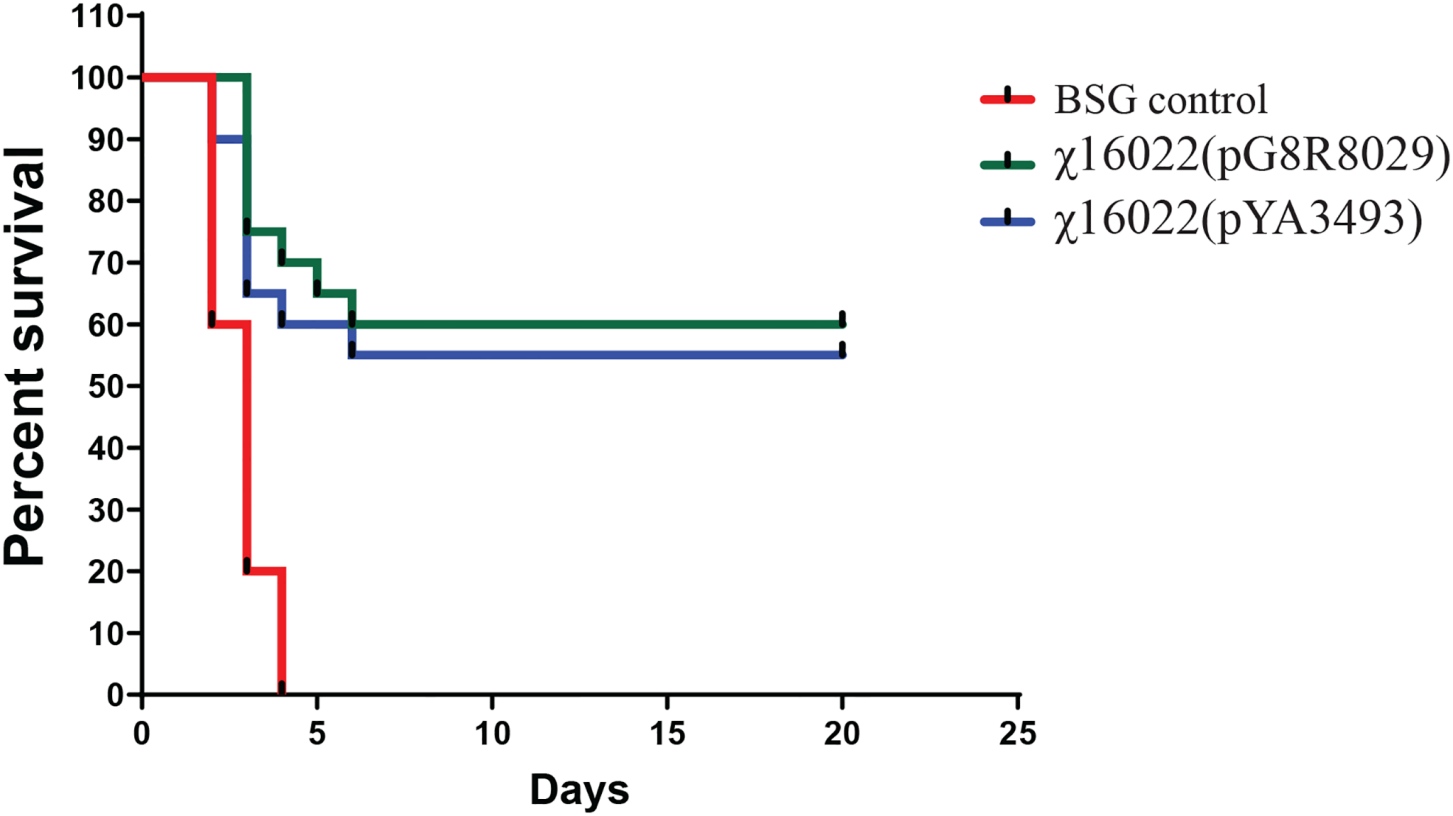
Survival of χ16022(pG8R8029), χ16022(pYA3493) vaccinated and BSG control fish after wild-type E. piscicida challenge. Control and 4 weeks post-vaccination zebrafish were i.c. challenged with 1 × 10^5^ CFU/dose (10 × LD_50_) of wild-type *E. piscicida.* Mortality was recorded daily and represented as percent survival.

In order to investigate the protective efficacy of χ16022(pG8R8029) in zebrafish against *E. piscicida* infection, the vaccinated and control fish were i.c. challenged with live virulent *E. piscicida* (J118) 1 x 10^5^ cells/fish (10 × LD_50_) at 4 weeks of post-vaccination. The mortality in fish started within 48 hours post *E. piscicida* infection (confirmed by isolating J118 from the kidney of dead fish). The vaccinated fish showed typical symptoms of *E. piscicida* infection such as hemorrhages of the skin and in internal organs and swelling of abdomen. Significantly greater survival was observed in the χ16022(pG8R8029)-vaccinated group (60%) and in the vector control χ16022(pY3493) group (55%) in comparison to that of the control unvaccinated fish (0%) over a period of 15 days post-challenge (**Figure 10**) against virulent *E. piscicida* strain J118.

## 4 Discussion

The advantage of using live bacterial vaccine vectors is their capability to invade and colonize internal tissues after mucosal delivery, which is critically important for inducing immune protection (32). Bacterial vaccine vectors have been used to produce protection against self as well as heterologous antigens (42–44). Our lab has developed a strategy of regulated-delayed attenuation in *Salmonella*, which makes the vaccine vectors safe and immunogenic for delivery of heterologous antigens to mice (45–47). We recently established the regulated-delayed attenuation phenotype in *E. piscicida* by using the arabinose-regulated promoter (5, 6). In this study, the promoters of both *fur* and *crp* genes were replaced with a tightly regulated *araC* P_araBAD_ cassette such that expression of these genes is dependent on arabinose which is provided during growth *in vitro*. Fur and Crp proteins cease to be synthesized due to the absence of arabinose and attenuation is progressively established *in vivo* to preclude induction of disease symptoms. Our study indicated that the *E. piscicida* strain carrying these two deletion insertion mutations is attenuated in zebrafish by i.c. injection.

To eliminate the need for an antibiotic-resistance selection marker for maintenance of plasmids in bacterial cells, our lab developed the balanced-lethal vector–host systems in *Salmonella* and *E. ictaluri* to ensure maintenance of the plasmid vectors *in vivo* and *in vitro*, without reliance on antibiotic-resistance markers (12, 14, 17). To develop a balanced-lethal system in *E. piscicida*, the *asdA* gene was characterized, whose product is essential for the synthesis of DAP, a crucial element of bacterial cell wall peptidoglycan. *E. piscicida* Δ*asdA* strain χ16000 can only survive when DAP is supplied in the growth media or contains a plasmid with the wild-type *asdA* gene. Since DAP is not present in *in vivo* environments, the Δ*asdA* strain will undergo lysis unless a plasmid with the *asdA* gene is present within the bacteria, establishing a powerful selection marker for maintenance of the plasmids within the RAEV, as observed previously in *Salmonella* and *E. ictaluri* (12, 14, 17). The ideal balanced-lethal system should have similar level of virulence as the wild-type strain. The amount of AsdA synthesis by the Asd^+^ vector in RASV affects the growth and immunogenicity of the strain (30). *E. piscicida* AsdA shares a high percentage of sequence identity and structural identity with, *E. ictaluri* (97.28%) and *Salmonella* (82.83%). As expected, plasmids containing the wild-type *asdA* gene from *E. ictaluri* and *Salmonella* were able to complement and establish the balanced-lethal system in *E. piscicida* Δ*asdA* strains. The pYA3493 AsdA^+^ vector system has been successfully used in live recombinant *Salmonella* and *E. ictaluri* vaccines (14, 30, 31, 48). Therefore, the pYA3493 AsdA^+^ vector system was used to deliver the antigens by RAEV. Vaccine efficacy depends on successful delivery of protein antigens for presentation in an optimal form and it has been observed that secreted proteins are immunogenic and interact with antigen-presenting cells because of their subcellular locations (49). β-Lactamase is a well-characterized periplasmic secreted protein in Gram-negative bacteria and translocation depends on β-Lactamase signal sequence (*bla* SS) composed of 23 amino acid (aa) N-terminal residues (50, 51). The pYA3493 plasmid contains the P_trc_ promoter region, β-lactamase signal sequence (*bla* SS), and a multicloning sites. It was designed for secretion of recombinant antigens to the periplasm from the cytoplasm (48).

Infection by *I. multifiliis* has been shown to elicit both primary and secondary immune responses in catfish that promote parasite clearance (21–24). This adaptive immune response in fish suggests that vaccination would be a safe and effective method to prevent white spot disease. Surface immobilization antigens (IAGs) expressed by *I. multifiliis* are immuno-dominant and have become candidate proteins for vaccine development (23). Channel catfish and rainbow trout immunized with DNA vaccines encoding IAG52B produced specific antibodies, but showed no significant increase in the level of protection in challenge studies, suggesting that naked DNA alone is insufficient to induce a strong enough immune response to confer protection (25, 26). We expect that the Ich antigen delivered by RAEV should solve this problem. Sera from immunized fish immobilize *I. multifiliis* theronts *in vitro* demonstrating a role for i-antigens in protective immunity (52). Cytokines play an important role in immune surveillance against bacterial infection in fish (53–55). It was observed that, RAEV-Ich strain induced cytokine *tnf-α*, *il-1β*, *il-6* and *il-8* gene expression in different tissues of zebrafish. Activation of proinflammatory cytokines is involved in regulating immunoglobulin synthesis in teleosts (5, 7, 56). Zebrafish receiving booster doses of a live attenuated *Vibrio anguillarum* vaccine at 2 weeks after primary vaccination were better protected against vibriosis in comparison to fish that received a single vaccination (57). In the current study, we delivered booster immunizations at 2 weeks after primary vaccination to ensure long-term protective immunity. We did not detect signs of disease in vaccinated fish during the immunization periods. Our study demonstrates that RAEV-Ich induced a mucosal and serum anti-IAG52B IgM titer in zebrafish. As expected, RAEV-Ich vaccine strain also induced *E. piscicida* protective immunity in the immunized zebrafish. Immunized fish showed elevated levels of *E. piscicida* anti-LPS IgM in both mucus and serum. The immune protection mediated by RAEV-Ich vaccine strain χ16022(pG8R8029) and vector-control strain χ16022(pYA3493) against *E. piscicida* infection were evaluated. The survival rate was higher than 60% and 55% in zebrafish immunized with χ16022(pG8R8029) and χ16022(pYA3493) respectively. This confirms that RAEV confers immune protection against the virulent *E. piscicida*.

In summary, we have invented and developed an innovative antibiotic-sensitive Recombinant Attenuated *Edwardsiella* Vaccine (RAEV) vector system with *in vivo* display of regulated-delayed attenuation. The *bla* SS-IAG52B fusion protein was synthesized and secreted into the RAEV periplasm and delivered it to fish. RAEV-Ich induced different levels of immune responses and gave significant protection to the fish against *E. piscicida* infection. RAEV-Ich also induces anti- IAG52B IgM in zebrafish suggesting this vaccine strain may protect fish against white-spot diseases. This multidisciplinary approach using cutting-edge technologies will address the sustainability challenges of aquaculture increased food security.

## Data Availability Statement

Publicly available datasets were analyzed in this study. This data can be found here: https://www.ncbi.nlm.nih.gov/genbank/CP001135.1, AAK94941.1.

## Ethics Statement

The animal study was reviewed and approved by University of Florida IACUC.

## Author Contributions

BS and RC contributed to conception funding acquisition and design of the study. BS wrote the first draft of the manuscript and contributed for the Methodology, Investigation, data analysis. CP wrote the sections of the manuscript, editing and methodology. All authors contributed to manuscript revision, read, and approved the submitted version.

## Funding

This work was supported by the grant of U.S. Department of Agriculture (USDA) - National Institute of Food and Agriculture – USDA-NIFA Grant No. 2018-67015-28286.

## Conflict of Interest

RC is a co-founder and part owner of Curtiss Healthcare, Inc., which is involved in developing vaccines against infectious diseases of farm animals.

The remaining authors declare that the research was conducted in the absence of any commercial or financial relationships that could be construed as a potential conflict of interest.

## Publisher’s Note

All claims expressed in this article are solely those of the authors and do not necessarily represent those of their affiliated organizations, or those of the publisher, the editors and the reviewers. Any product that may be evaluated in this article, or claim that may be made by its manufacturer, is not guaranteed or endorsed by the publisher.
